# Expression of Lectin-Like Transcript 1, the Ligand for CD161, in Rheumatoid Arthritis

**DOI:** 10.1371/journal.pone.0132436

**Published:** 2015-07-06

**Authors:** Paulina Chalan, Johan Bijzet, Minke G. Huitema, Bart-Jan Kroesen, Elisabeth Brouwer, Annemieke M. H. Boots

**Affiliations:** 1 Department of Rheumatology and Clinical Immunology, University of Groningen, University Medical Center Groningen, Groningen, The Netherlands; 2 Department of Laboratory Medicine, University of Groningen, University Medical Center Groningen, Groningen, The Netherlands; University of Lisbon, PORTUGAL

## Abstract

**Objectives:**

Precursor Th17 lineage cells expressing CD161 are implicated in Rheumatoid Arthritis (RA) pathogenesis. CD4+CD161+ T-cells accumulate in RA joints and may acquire a non classical Th1 phenotype. The endogenous ligand for CD161 is lectin-like transcript 1 (LLT1). CD161/LLT1 ligation may co-stimulate T-cell IFN-γ production. We investigated the presence and identity of LLT1-expressing cells in RA synovial fluid (SF) and synovial tissue (ST). We also assessed levels of soluble LLT1 (sLLT1) in different phases of RA development.

**Methods:**

Paired samples of peripheral blood mononuclear cells (MC) and SFMC (n = 14), digested ST cells (n = 4) and ST paraffin sections (n = 6) from late-stage RA were analyzed for LLT1 expression by flow cytometry and immunohistochemistry. sLLT1 was measured using a sandwich ELISA. Sera and SF from late-stage RA (n = 26), recently diagnosed RA patients (n = 39), seropositive arthralgia patients (SAP, n = 31), spondyloarthropathy patients (SpA, n = 26) and healthy controls (HC, n = 31) were assayed.

**Results:**

In RA SF, LLT1 was expressed by a small proportion of monocytes. In RA ST, LLT1-expressing cells were detected in the lining, sublining layer and in areas with infiltrates. The LLT1 staining pattern overlapped with the CD68 staining pattern. FACS analysis of digested ST confirmed LLT1 expression by CD68+ cells. Elevated systemic sLLT1 was found in all patient groups.

**Conclusions:**

In RA joints, LLT1 is expressed by cells of the monocyte/macrophage lineage. Serum levels of sLLT1 were increased in all patient groups (patients with early- and late-stage RA, seropositive arthralgia and spondyloarthropathy) when compared to healthy subjects.

## Introduction

Human T lymphocytes expressing killer cell lectin-like receptor CD161 (NKR-P1A) have gained increased appreciation over the last decade. CD161+ T-cells were identified as precursor Th17 cells involved in chronic auto-inflammatory disorders. Specifically, CD161+ Th17-lineage cells were implicated in the pathogenesis of Crohn's disease [1], giant cell arteritis [2] and psoriasis [3] by accumulation and active IL-17 expression in the disease-affected sites. CD161+ cells can acquire a non classical Th1 phenotype, manifested by IFN-γ and T-bet expression, thought to be driven by IL-12 at the site of inflammation [4,5]. CD161+Th1 cells have been reported to accumulate in the synovial fluid (SF) of juvenile idiopathic arthritis (JIA) [5] and rheumatoid arthritis (RA) patients [6].

The sole endogenous ligand for CD161 is lectin-like transcript 1 (LLT1) [7,8]. Despite the growing evidence supporting a role of CD161+ T-cells in autoimmune pathology, no studies have yet addressed the expression of lectin-like transcript 1 in autoimmune conditions.

Human LLT1 is a product of the CLEC2D gene belonging to the C-type lectin domain family 2 (CLEC2) of the C-type lectin-like receptors (CTLR), which also includes CLEC2A (keratinocyte- associated C-type lectin, KACL), CLEC2B (activation- induced C-type lectin, AICL) and CLEC2C (CD69) [9,10]. Surface expressed LLT1 represents isoform 1 of the CLEC2D gene generated via alternative RNA splicing [11] and is the only protein isoform for which the ability to bind CD161 has been confirmed [12]. CLEC2D isoforms 2 and 4 are expressed as transmembrane proteins residing in the endoplasmic reticulum. Alignment of the predicted CLEC2D protein isoforms identified alternative splicing variant 5 as a putative soluble form of LLT1 [11]. Circulating leukocytes are characterized by low LLT1 expression at both the mRNA [11,13] and protein level [13]. LLT1 upregulation has been associated with the activation status of the cell. Surface-expressed LLT1 was detected in stimulated T-cells, B-cells and NK-cells. LLT1 was not found on circulating monocytes or immature monocyte-derived dendritic cells (DC) but became upregulated on TLR-activated mature monocyte-derived and plasmacytoid DC [13,14].

Previously, the LLT1-CD161 interaction was reported to co-stimulate T-cell effector functions and to enhance IFN-γ production (a feature associated with the Th1 phenotype) [7]. Our previous findings showing enrichment of CD4+CD161+ T-cells at the local inflammatory site and their local skewing towards the Th1 phenotype [6], prompted us to investigate whether LLT1 is upregulated in the pro-inflammatory environment of the disease-affected joints. We aimed to define which antigen presenting cells (APC) may participate in the crosstalk with CD161+ T-cells by analyzing LLT1 expression on different immune cell populations from synovial fluid and synovial tissue (ST) of late-stage RA patients. In addition, we hypothesized that LLT1 may be expressed as a soluble protein. We therefore assessed not only the presence of surface-expressed LLT1 but also its soluble form in the serum and synovial fluid from patients in different stages of disease. In addition, sera from seropositive arthralgia patients (SAP) who are at risk of developing RA [15,16] and patients with spondyloarthropathy (SpA), were included in this analysis.

## Materials and Methods

### Study participants

Forty-four long-standing, treated RA patients; 54 recently diagnosed RA patients; 30 patients seropositive for anti-CCP and/or RF with (a history of) arthralgia (SAP), 26 patients with spondyloarthropathy (SpA) and 31 healthy controls were included in the present study (Table 1). Absence of arthritis in SAP was confirmed by physical examination of 44 joints by a trained senior rheumatologist (EB). SAP were treated with various non-steroidal anti-inflammatory drugs (NSAIDs) only. Fifty-four patients included in the early RA group had their blood drawn at diagnosis before start of treatment with disease modifying anti-rheumatic drugs (DMARD). Long-standing RA patients received, DMARDs, biologicals, NSAIDs and glucocorticoids. Most SpA patients received NSAIDs and only few received DMARDs and/or glucocorticoids. HC volunteers were found among employees at the University Medical Center Groningen. All RA patients fulfilled 1987 or 2010 American College of Rheumatology (ACR) classification criteria for RA. Written informed consent was obtained from all participants for use of their samples in biomarker research. All procedures were in accordance with institutional guidelines and approved by the local ethics committees of the University Medical Center Groningen (UMCG) and Medical Center Leeuwarden (MCL) [6,17].

**Table 1:** Clinical and demographical characteristics of the subjects included in the study.

	HC	SAP	Early RA	Late RA	SpA
N	31	30	54	44	26
Age [yrs]; mean (SD)	54.6 (7.5)	50.4 (14.5)	58.0 (13.2)	51.8 (11.8)	42.4 (13.4)
Gender; % female	67.7	66.7	77.8	65.9	53.9
CRP [mg/l]; median (range)	nd	5.0 (5.0–29.0)	14.5 (5.0–108.0)	26.0 (4.0–185.0)	9.0 (3.0–48.0)
ESR [mm/h]; median (range)	nd	12.0 (2.0–69.0)	28.0 (2.0–96.0)	33.0 (2.0–120.0)	14.0 (2.0–48.0)
DAS28; mean (SD)	na	na	4.9 (1.4)	4.9 (1.7)	na
RF; % positive (n)	nd	80.0 (24)	70.4 (38)	77.3 (34)	0.0 (0)
Anti-CCP; % positive (n)	nd	90.0 (27)	74.1 (40)	81.4 (35)	11.5 (3)
BASDAI; mean (SD)	na	na	na	na	5.0 (2.3)
ASDAS; mean (SD)	na	na	na	na	3.2 (1.1)

HC = healthy controls; SAP = seropositive arthralgia patients; RA = rheumatoid arthritis; SpA = spondyloarthropathy; CRP = C-reactive protein; ESR = erythrocyte sedimentation rate; DAS28 = disease activity score 28; RF = rheumatoid factor; anti-CCP = anti-cyclic citrullinated proteins antibodies; BASDAI = bath ankylosing spondylitis disease activity index; ASDAS = ankylosing spondylitis disease activity score; nd = not defined; na = not applicable.

Mononuclear cells derived from peripheral blood (PBMC) and synovial fluid (SFMC) from 14 out of 44 long-standing RA patients were used for FACS analysis of LLT1 surface expression.

To study surface-expressed LLT1 at the site of inflammation, 6 synovial tissue biopsies obtained from hand, shoulder or knee joints from long-standing, treated RA patients who underwent joint replacement surgery or synovectomy were processed for immunohistochemistry. In addition, ST biopsies obtained from 4 late-stage RA patients undergoing hip (n = 1) or knee (n = 3) joint replacement surgery were enzymatically digested for cell isolation.

To assess the presence of soluble LLT1, paired serum and synovial fluid samples from 26 out of 44 long-standing RA as well as serum samples from 40 out of 54 early RA patients; all 30 SAP; 31 HC and 26 SpA patient sera were assayed.

### Detection of LLT1-bearing leukocyte subsets in paired PBMC and SFMC

Following thawing of cryopreserved PBMC and SFMC, cells were resuspended at a concentration of 1x10^6^ cells/100 μl in PBS with 10% heat-inactivated human AB serum (CTL-Europe GmbH, Bonn, Germany) to block surface Fc receptors. The cell suspension was incubated for 30 min at room temperature (RT) with the following mouse monoclonal anti-human antibodies: CD3 PerCP (Cat No 347344, BD Bioscience, Breda, The Netherlands), CD56 APC-H7 (Cat No 302216), CD16 AlexaFluor700 (Cat No 302026, BioLegend, San Diego, CA, USA), LLT1 APC (clone 402659, Cat No FAB3480A, R&D Systems, Abingdon, UK), CD14 eFluor 605NC (Cat No 93–0149, eBioscience, Vienna, Austria). Cells were analyzed using LSR II flow cytometer (BD Biosciences) and data analysis was performed with Kaluza analysis software (Beckman Coulter, Woerden, the Netherlands).

### Immunohistochemical detection of LLT1 in rheumatoid synovial tissue

Mouse monoclonal anti-human CLEC2D antibody (clone 4C7, Cat No H00029121-M01, Abnova, Taipei City, Taiwan) was used for the detection of LLT1 in the synovial tissues (diluted 1:100). To further characterize LLT1- expressing cells, consecutive tissue sections were stained with the following antibodies: mouse monoclonal against human macrophage marker CD68 (IgG3, Cat No M0876, diluted 1:100), rabbit polyclonal against human T-cell marker CD3 (Cat No A0452,diluted 1:300), mouse monoclonal against human B-cell marker CD20cy (IgG2a, Cat No M0755, diluted 1:50, all from Dako, Glostrup, Denmark). Briefly, 5 μm paraffin sections were deparaffinized and rehydrated using the standard procedures, followed by microwave heating in Tris-EDTA buffer (pH 9.0) at 99°C for 30 min in order to retrieve the antigens. After cooling and washing in three fresh changes of phosphate-buffered saline (PBS) for 5 min each, blocking of tissue endogenous peroxidase was performed by incubation with 0.1% hydrogen peroxide in PBS for 30 min. Avidin/ Biotin blocking kit was used according to the manufacturer’s protocol (Vector Labs, Burlingame, CA, USA). Non-specific antibody binding was blocked by incubating the sections with PBS containing 20% goat serum or 2.5% horse serum for 20 min. Following washing with PBS, tissue sections were incubated with the primary antibodies diluted in PBS with 1% bovine serum albumin (BSA; Sigma-Aldrich, Zwijndrecht, the Netherlands) for 60 min at room temperature, washed with PBS and the following secondary antibodies reagents were used: anti-mouse Ig- alkaline phosphatase and mouse anti-alkaline phosphatase (APAAP) detection kit (Dako); goat anti-mouse IgG3- horseradish peroxidase (HRP) antibody (diluted 1:50; SouthernBiotech, Birmingham, AL, USA); anti-rabbit Ig- Peroxidase detection kit (Vector Labs) or goat anti-mouse IgG2a-HRP antibody (diluted 1:50). DAB substrate- chromogen system (Dako) or Fast Red phosphate substrate (APAAP detection kit) were used according to the manufacturer’s instructions.

Pictures of 3–5 different areas of each section were semi-quantitatively scored by three independent researchers (PC, JB, MH) with the number of positive cells ranked on a 4-point scale: 0 = none; 1 = <5%; 2 = 5–50%; 3 = >50%. Within each area lining layer, sublining layer, vessels and infiltrates were scored separately for the expression of all markers used for the immunohistochemical staining. The synovitis (Krenn) score was calculated as described elsewhere [18]. Briefly, the synovitis score was defined as the sum of the score of the lining layer enlargement (from 0 [1 layer of lining cells] to 3 [> 5 layers of lining cells]), the score of the synovial stroma cell density (from 0 [normal cellularity] to 3 [greatly enhanced cellularity]) and the score of the inflammatory infiltrate (from 0 [no inflammatory infiltrate] to 3 [dense band-like infiltrate or numerous follicle-like infiltrates]). Obtained Krenn score indicates absence of synovitis when 0–1, low-grade synovitis when 2–4 or high-grade synovitis when 5–9.

### Synovial tissue digestion and analysis

Cells were isolated from ST biopsies, obtained from 4 late-stage RA patients as described [6]. Cells were resuspended in RPMI-1640 containing 10% fetal bovine serum (FBS; Lonza, Breda, the Netherlands), 10% dimethyl sulfoxide (DMSO) and stored in liquid nitrogen until analysis. ST cells from all 4 subjects were thawed at the same time and were stained with Fixable Viability Stain 450 (BD Biosciences) according to the manufacturer’s instructions. Following washing with PBS containing 0.5% BSA and 2 mM EDTA, cells were resuspended in the washing buffer with Fc receptors blocking reagent (Miltenyi Biotech) and stained with the following mouse monoclonal anti-human antibodies: LLT1 PE (clone 402659, Cat No FAB3480P, R&D Systems), CD3 eFluor 605NC (Cat No 93–0037), CD19 PE-Cyanine7 (Cat No 25–0199, both from eBioscience), CD161 APC (Cat No 130-092-678, Miltenyi Biotech) for 30 min. at RT. After fixation and permeabilization with the Foxp3/Transcription Factor Staining Buffer Set (eBioscience), cells were stained with mouse monoclonal anti-human CD68 FITC antibody (Cat No 11–0689, eBioscience) for 30 min. at RT, followed by washing with the permeabilization buffer (eBioscience). ST cells were analyzed using LSR II flow cytometer (BD Biosciences), and data analysis was performed with Kaluza analysis software (Beckman Coulter).

### Detection of soluble LLT1 using ELISA

Serum samples were used to determine levels of sLLT1 with anti-CLEC2D/OCIL/LLT1 sandwich ELISA (Cat No MBS936829, MyBioSource, San Diego, CA, USA) according to the manufacturer's instructions. In order to assess the impact of RF interference on the assay performance, we investigated whether RF is bound by the anti-LLT1 detection antibody of the ELISA kit. For that purpose 16 RF-positive serum samples (12/16 positive for IgA-RF [range 45–3000 U/ml], 9/16 positive for IgG-RF [range 106–32 U/ml], 13/16 positive for IgM-RF [range 372–26 U/ml] were used. Fc-free RF was obtained for 8 out of 16 serum samples by incubation for 22 h at 37°C with pepsin (Sigma-Aldrich), diluted in sodium acetate buffer (0.1 M, pH 3.6) to a final concentration of 0.67 mg/ml. RF-deprived and untreated serum samples were incubated on a rabbit IgG-coated plate used for RF measurement (IBL International, Hamburg, Germany). After washing, anti-LLT1 detection antibody was added to the plate and the assay was continued according to the manufacturer’s instructions. We observed no RF binding by the anti-LLT1 detection antibody (OD values <0.4). RF removal did not cause further reduction of the observed OD values (data not shown).

### Statistical analysis

Statistical analysis was performed with GraphPad Prism version 5.0 (GraphPad Software, San Diego, CA, USA). Normally distributed data were analyzed using unpaired t test. Non-normally distributed data were analyzed using Mann-Whitney 2-tailed test. Paired sample analysis was performed with Wilcoxon signed rank test. P<0.05 was considered statistically significant.

## Results

### Surface-expressed LLT1 detected on a proportion of synovial fluid monocytes

**Fig 1:**
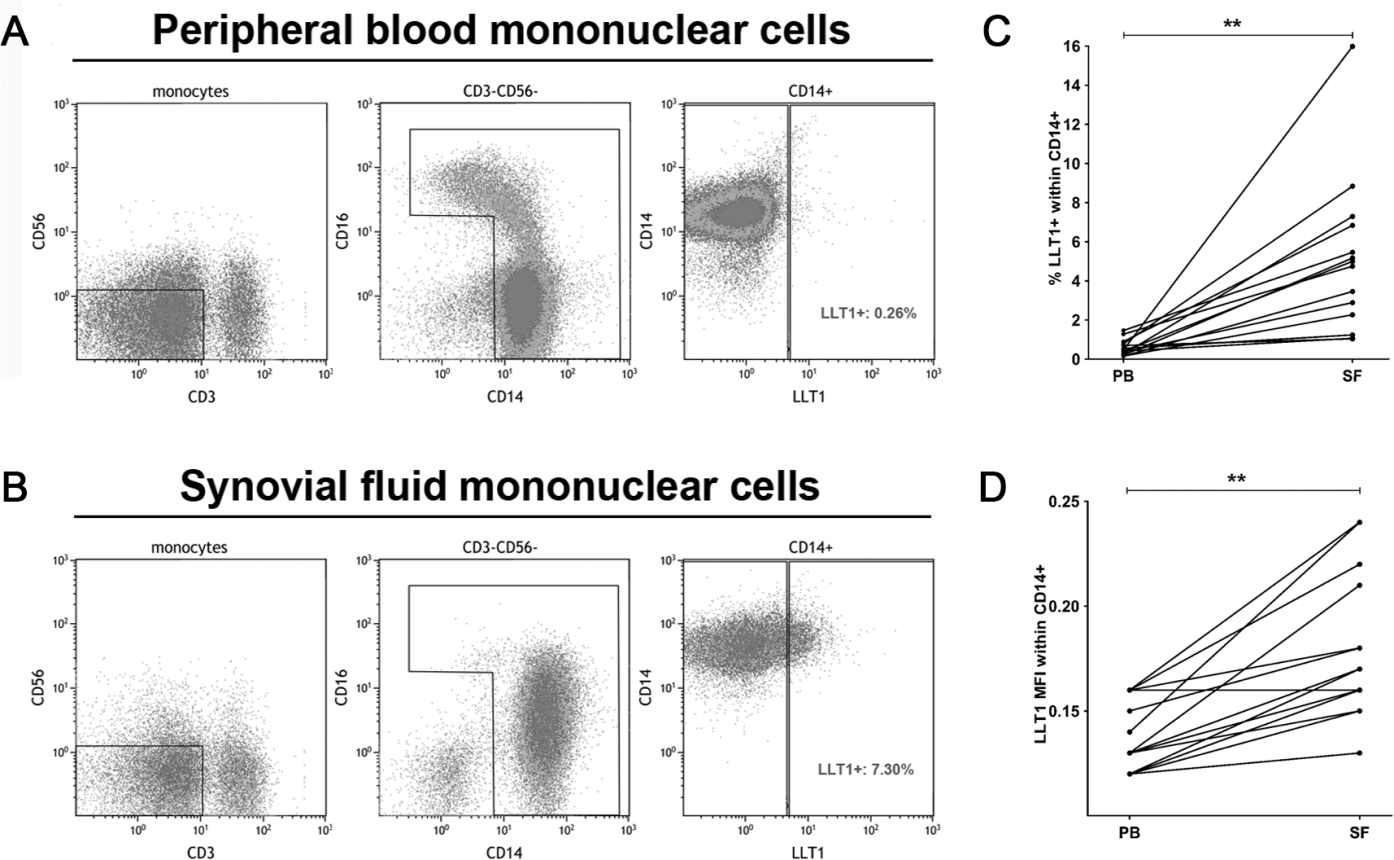
Surface-expressed LLT1 is found on SF monocytes. Monocytes from A) peripheral blood and B) synovial fluid were gated based on forward and side scatter characteristics. After excluding CD3+ and CD56+ lymphocytes, monocytes were gated based on CD14 and CD16 expression. The frequency of LLT1+ cells was assessed within the total monocyte population. C) The frequency of LLT1+ monocytes and D) LLT1 MFI from paired samples of PB and SF (n = 14). Mouse monoclonal anti-LLT1 antibody, clone 402659 (R&D Systems) was used.

To investigate if LLT1 is upregulated in the pro-inflammatory environment of arthritic joints we first analyzed paired SFMC and PBMC from late-stage RA for LLT1 surface expression (Fig 1A–1D). Flow cytometric analysis detected LLT1 expression on a proportion of SF-derived monocytes whereas LLT1 was hardly detected on PB monocytes (median 5% [range 1.0–16.0%] vs 0.4%, [range 0,2–1,5%] of the CD14+ monocyte population) (Fig 1A, 1B and 1C). LLT1 mean fluorescence intensity (MFI) was also significantly increased within the SF-derived monocytes compared to PB monocytes (Fig 1D). In agreement with previous reports [11,13], we did not detect expression of LLT1 by circulating T-cells, B-cells, NK-cells, monocytes and granulocytes in PB samples of newly diagnosed RA or healthy controls (data not shown).

### LLT1- expressing cells detected in RA synovial tissue

**Fig 2:**
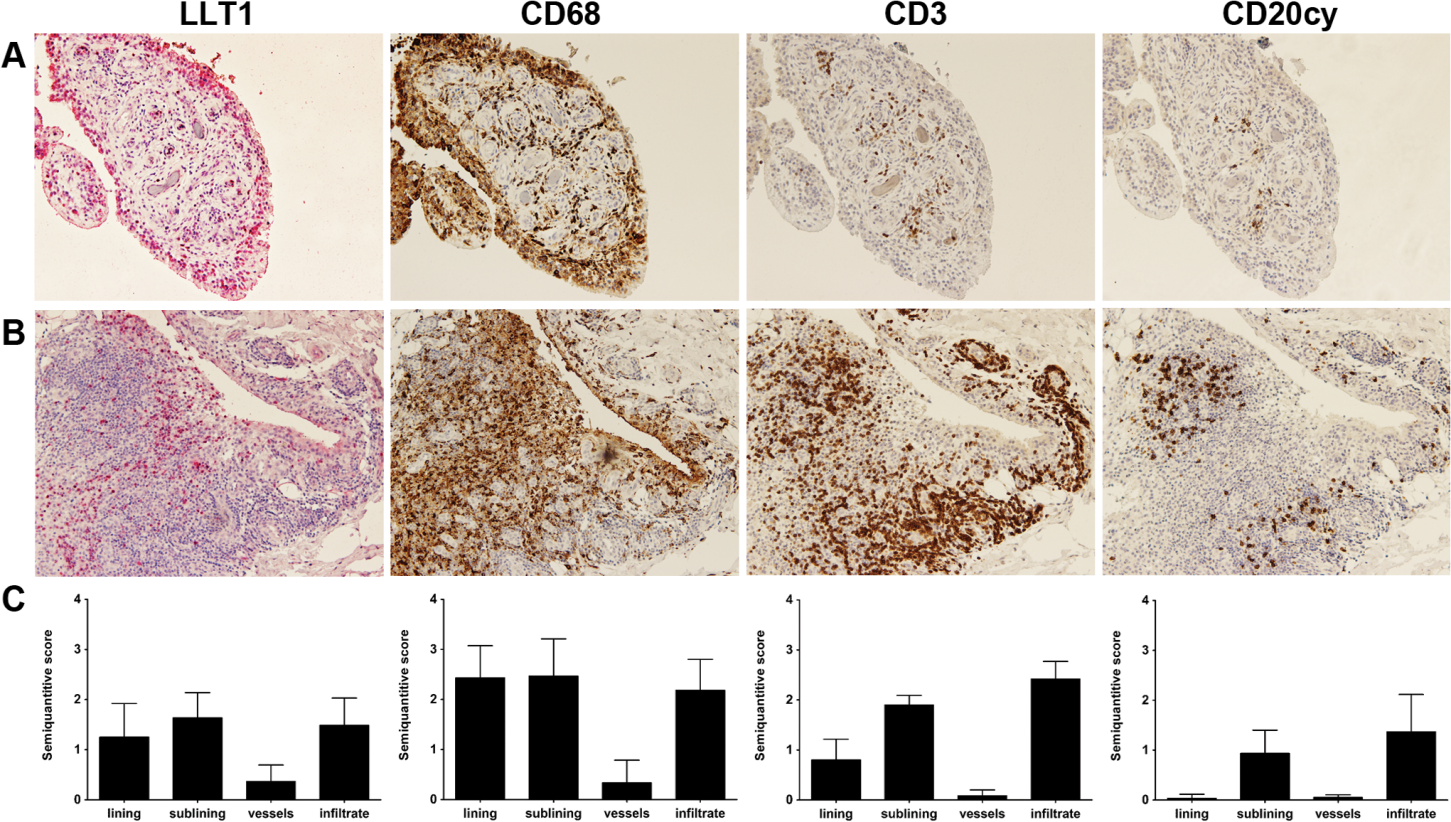
Immunohistochemical detection of LLT1 expression in RA ST cells. To study surface-expressed LLT1 at the site of inflammation, 6 synovial tissue biopsies obtained from hand, shoulder or knee joints from long-standing, treated RA patients who underwent joint replacement surgery or synovectomy were processed for immunohistochemistry. Representative pictures showing immunohistochemical staining of consecutive tissue slides from 2 late-stage RA patients stained with antibodies against LLT1, CD68, CD3 and CD20cy (A,B). Graphs in C depict the results of the semiquantitative scoring (mean + SD) performed by 3 independent researchers. Scoring of the staining of all the markers was performed according to a 4-point scale: 0 = no positive cells; 1 = <5% positive cells; 2 = 5–50% positive cells; 3 = >50% positive cells. Three to five different pictures of each slide section (n = 6 different sections per biopsy) were taken. In each picture the lining, sublining, lymphoid infiltrate area’s (defined based on CD3 and CD20cy staining) and blood vessels were scored separately for the expression of LLT1, CD68, CD3 and CD20cy. Mouse monoclonal anti-LLT1, clone 4C7 (Abnova) was used.

Following the detection of surface LLT1 by a proportion of SF monocytes, we next investigated the presence of LLT1-bearing cells in synovial tissue. To that end, synovial tissue specimens with low- to high-grade synovitis (median Krenn score of 5 [range 2–7]) [18] were obtained from six RA patients. Consecutive sections of synovial tissue were stained with antibodies against LLT1, CD68, CD3 and CD20cy (Fig 2). LLT1 staining was detected in all six biopsies and was predominant in the lining layer which showed pathological enlargement in all 6 biopsies analyzed (≥1 point according to Krenn et al [18]). LLT1 staining was also found in the sublining and in transitional areas consisting of different cell types [19]. Small numbers of LLT1+ cells were observed in scattered lymphoid infiltrates and more dense perivascular infiltrates. These synovial membrane areas were defined based on CD3 (T-cells) and CD20cy (B-cells) staining. Analysis of the results of the semi-quantitative scoring showed that 3/6 tissue biopsies had low (median score 1, indicating <5% positive cells) and 2/6 biopsies had moderate (median score 2, indicating 5–50% positive cells) expression of LLT1 in the lining layer. LLT1 expression in the sublining layer was found to be low (median score 1) in 4/6 and moderate (median score 2) in 2/6 biopsies while the infiltrate showed low and moderate LLT1 expression in 3/6 and 2/6 biopsies, respectively. In addition, some LLT1 staining of blood vessels was observed. The LLT1 staining pattern showed a marked overlap with CD68 staining. In all biopsies analyzed, CD68 expression was predominant in the lining and sublining layer and to a lesser extent in the lymphoid infiltrate areas.

**Fig 3:**
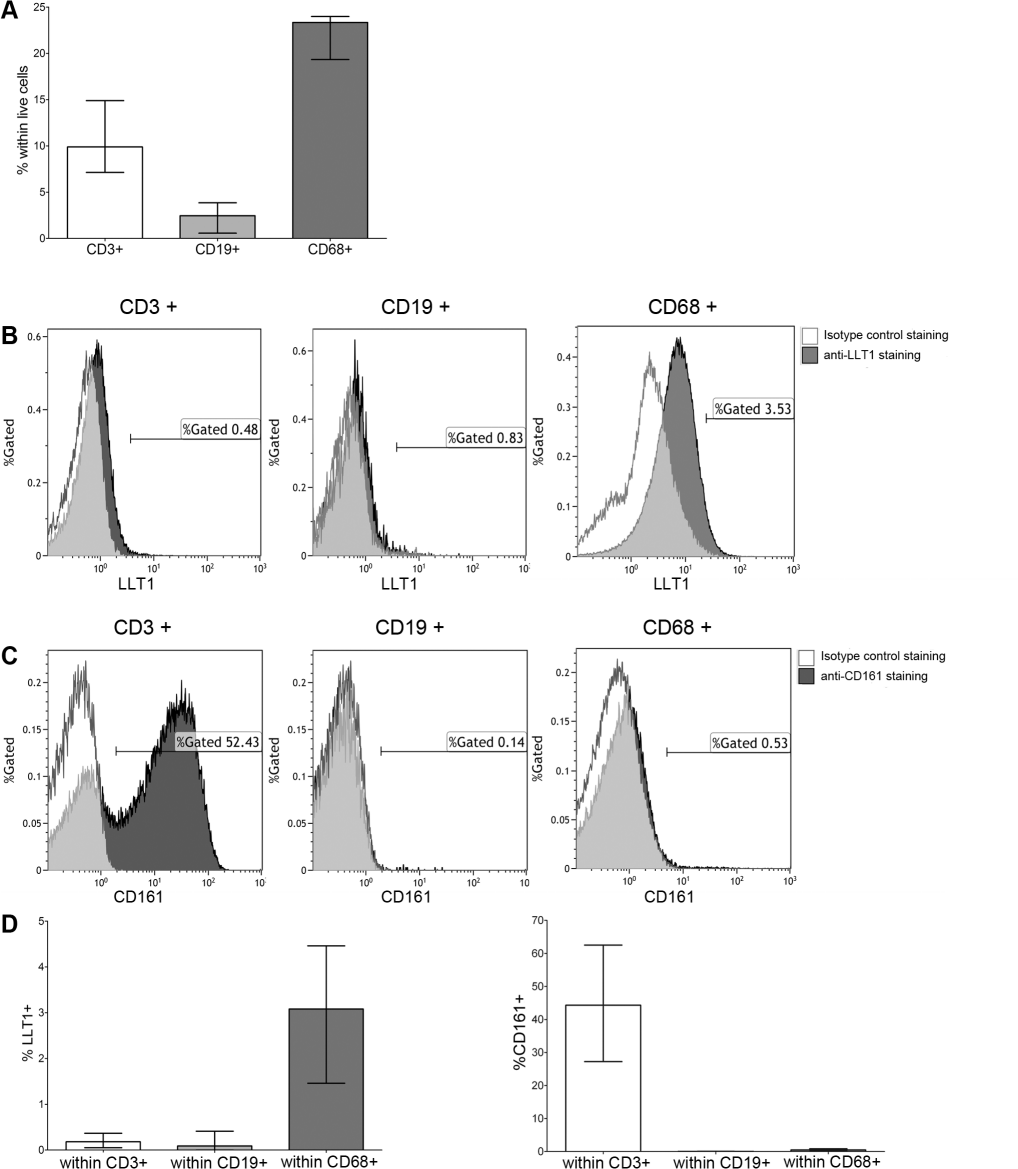
Flow-cytometric detection of LLT1 expression in RA ST cells. A) Percentages of CD3+ T-cells, CD19+ B-cells and CD68+ macrophages detected within the live cells gate of digested ST cells with flow cytometry. Briefly, necrotic cells were gated out based on the staining with the Fixable Viability Stain dye. Within the live cells gate lymphocytes and macrophages were gated based on FSC/SSC characteristics and CD68 expression, respectively. Within the lymphocyte gate T-cells and B-cells were gated based on CD3 and CD19 expression, respectively. Representative histogram overlays showing frequencies of B) LLT1+ and C) CD161+ cells within the populations of CD3+, CD19+ or CD68+ cells when compared to isotype control. D) Graphs show the percentages of LLT1 and CD161+ cells. Data from 4 independent donors were pooled. Bars represent the median value ± interquartile range. Mouse monoclonal anti-LLT1 antibody, clone 402659 (R&D Systems) was used.

To further confirm LLT1 expression by synovial macrophages, we performed a flow cytometric analysis of LLT1 expression on cells derived from digested RA synovial tissue biopsies (n = 4). The digested ST cellular composition constituted mainly CD68+ cells, T-cells and few B-cells (Fig 3A). LLT1 expression was upregulated by ST CD68+ macrophages as evidenced by a shift in LLT1 mean fluorescence intensity (MFI, Fig 3B). In contrast, synovial tissue derived B- and T-cells were found to be LLT1 negative (Fig 3B and 3D). As before, a high percentage of CD161+ T-cells were detected [6]. CD161 expression was not detected on B-cells or on CD68+ macrophages (Fig 3C and 3D). Thus, we conclude that in the ST, LLT1 and CD161 are expressed *in trans* by CD68+ macrophages and CD4+ T-cells, respectively (Fig 3D). This would allow potential crosstalk of LLT1-bearing APC with CD161+ T-cells.

### Soluble LLT1 levels are elevated in sera from SAP, early and late-stage RA patients

**Fig 4:**
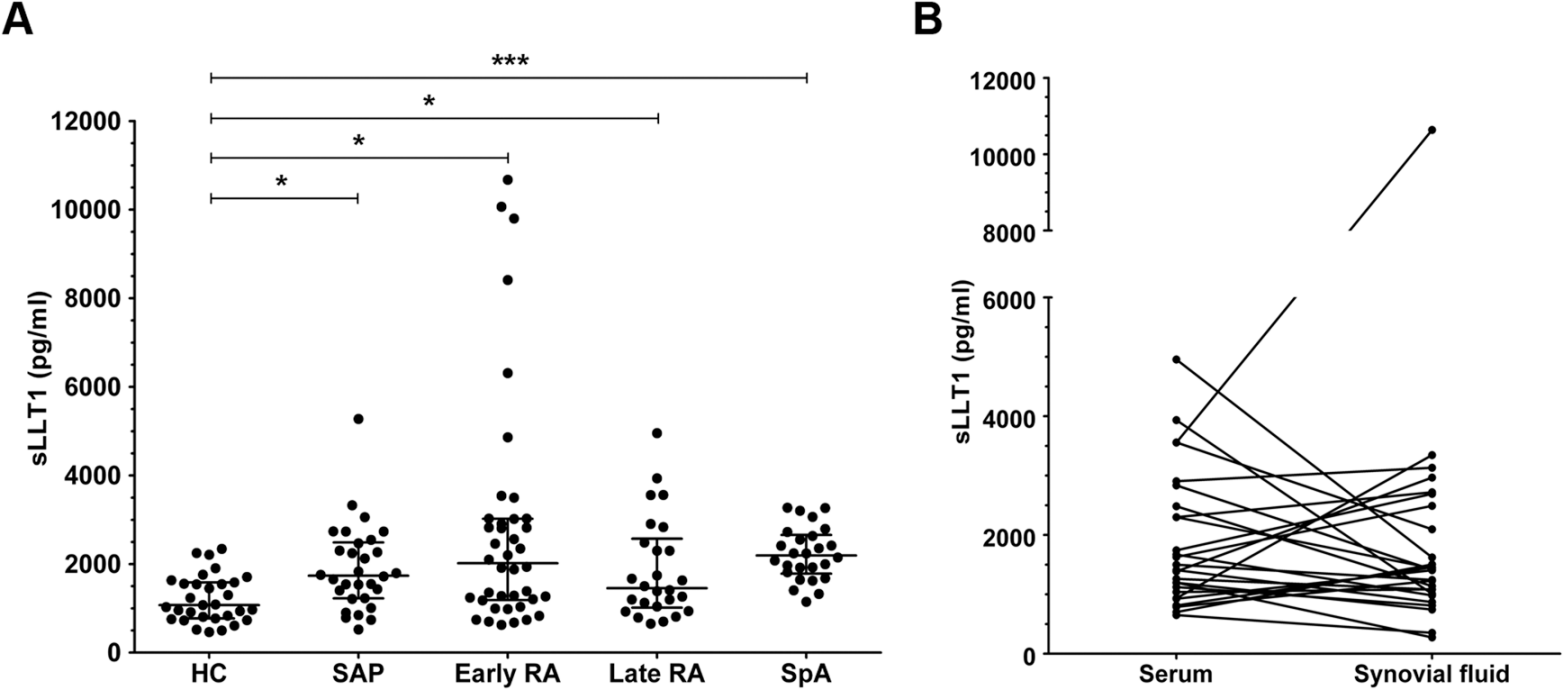
sLLT1 is increased in the serum of SAP, early and late-stage RA and SpA patients. A) Sera from HC (n = 31), SAP (n = 31), early RA patients (n = 39) and late RA patients (n = 26) and SpA patients (n = 26) were used to quantify the levels of soluble LLT1 using sandwich ELISA. Horizontal lines represent the mean value. Unpaired t test was used. B) Paired SF samples were used to compare the level of soluble LLT1 in PB and SF of long-standing RA (n = 26; Wilcoxon matched pairs test). Statistical significance is indicated as * for p <0.05, ** for p <0.001, and *** for p <0.0001. Rabbit polyclonal anti-LLT1 antibodies provided with a commercially available ELISA (MyBiosource) were used.

sLLT1 was detected in the sera of SAP, early and late-stage RA patients using sandwich ELISA. Systemic LLT1 values ranged from 275 pg/ml to 10675 pg/ml. All patients groups, including SAP (mean sLLT1 level 1909 pg/ml [SD = 971]), early RA (2813 pg/ml [2636]) and late-stage RA (1877 pg/ml [1137]) were characterized by significantly higher levels of sLLT1 compared to HC (1216 pg/ml [533]). To test if elevated sLLT1 is linked to the presence of autoantibodies and/or RF, we also tested sera from patients with RF-negative spondyloarthropathy (SpA, n = 26). Here the levels of sLLT1 were also significantly increased when compared to healthy controls (mean sLLT1 level 2225 pg/ml [SD = 597]; Fig 4A). Further, in late-stage RA, we did not detect an increase of sLLT1 in the synovial fluid when comparing paired SF and PB samples (Fig 4B). The data suggest a systemic rather than a local elevation of sLLT1. Serum levels of LLT1 were not correlated with measures of general inflammation (CRP and ESR), RA disease activity (DAS28) or SpA disease activity (BASDAI and ASDAS; data not shown). We also did not observe a correlation between sLLT1 and disease duration (data not shown).

## Discussion

This is the first study showing that surface-expressed LLT1 is present at the site of local inflammation in RA. The finding of LLT1 expression by cells of the monocyte/macrophage lineage in RA joints suggests potential crosstalk with CD161+ T-cells.

We aimed to identify which APCs express LLT1 at the level of the joint in RA. In late-stage RA, we investigated both the synovial fluid and the synovial tissue for the presence of LLT1 expressing cells. In SF, LLT1 expression was found in a small proportion of CD14+ monocytes. In RA synovial tissue, LLT1+ cells were found primarily in the lining layer enriched with macrophages [20] as evidenced by CD68 staining. It has been reported that the anti-LLT1 mouse antibody used for the tissue staining (clone 4C7) recognizes the CLEC2A isoform as well [11]. To confirm the detection of LLT1 in RA synovial tissue we performed a flow cytometric analysis of the digested ST using anti-LLT1 antibody (clone 402659) which is specific for LLT1. This antibody does not bind isoforms 2 and 4 of the CLEC2D gene nor other members of the CLEC2 family [11]. The combined data confirm that LLT1 is expressed by CD68+ ST macrophages.

We found LLT1 expression in RA joints to be confined to cells of the monocyte/macrophage lineage only. This may be explained by the notions that 1) LLT1 expression can be induced in B-cells and DCs following their *in vitro* stimulation with TLR ligands [13,14], 2) the expression of several TLR ligands [21–23] was previously found to be increased at RA inflammatory sites, 3) several pro-inflammatory cytokines (particularly Th1 cytokines such as IFN-γ) implicated in RA pathogenesis amplify the TLR-induced cellular activation, 4) T-cells [24], B-cells [25], monocytes [26] and neutrophils [27] in RA SF are characterized by an activated phenotype.

The presence of LLT1-bearing APC at the site of inflammation in RA may suggest their interaction with CD161+ Th17 lineage cells. Previous studies, including our own, have demonstrated the accumulation of CD4+CD161+ cells in RA joints where they show a non-classical Th1 phenotype [4,6]. Synovial fluid-derived IL-12 has been implicated in Th1 skewing [4]. CD161-triggering has been demonstrated to induce IFN-γ expression [7,13]. CD161 has a co-stimulatory role, thus the simultaneous engagement of TCR and CD161 is predicted to enhance T-cell function [7,28]. This suggests the contribution of the CD161-LLT1 co-stimulatory pathway to Th1 skewing at the level of RA joint. Our data demonstrate that the *in trans* expression of LLT1 and CD161 by CD68+ macrophages and CD4+ T-cells respectively, may allow for this intercellular (APC-T-cell) communication. Apart from their presence in the synovial lining and sublining, LLT1+ APC were also found in diffuse cellular infiltrates and perivascular lymphoid infiltrates where the presence and proximity of T- and B-lymphocytes were also demonstrated. Direct contact between T-cells and macrophages in RA synovium was demonstrated previously [29]. Technical limitations, however, prohibited a clear visualization of LLT1-CD161 interacting cells at the level of the ST. This was caused by incompatibility of the CD161 and LLT1 antibodies in different IHC protocols. More studies are thus required to formally prove that these cells interact locally and to prove that LLT1-CD161-mediated co-stimulation contributes to Th1 skewing at the level of the joint in RA.

Our studies also revealed LLT1 staining of the ST blood vessels. The data suggest a facilitating role for the LLT1-CD161 interaction in transendothelial migration of CD4+CD161+ T-cells to inflammatory sites.

In this report we show for the first time that soluble LLT1 can be detected in the peripheral blood and that sLLT1 levels are increased in autoinflammatory conditions when compared to the non-diseased state. Soluble LLT1 was found to be elevated in the sera of RA (irrespective of the disease stage), arthralgia patients who are at risk of developing RA [15,16] and patients with spondyloarthropathy. SpA is regarded as an autoinflammatory rather than an autoimmune rheumatic disease [30]. Similar to other proteins from the same C-type lectin-like domain 2 family, such as CLEC2A [31], AICL [32] and CD69 [33], LLT1 expression has been associated with the cellular activation status [11,13,34]. Its rapid upregulation by various cell types including T-cells, B-cells, NK-cells suggests that LLT1 may be an universal and early activation marker. In agreement with others [13] we did not observe surface LLT1 expression on ex vivo analyzed circulating immune cells. Increased levels of sLLT1 could indicate a prompt shedding following cell activation. A similar phenomenon has been reported for soluble CD25 (sIL-2R) which is released upon cell activation [35]. sCD25 was found to be increased in the periphery of patients with chronic autoimmune diseases including RA [36]. A recent study provided evidence for LLT1 upregulation by non-hematopoietic cells (e.g. epithelial cells) in response to pro-inflammatory cytokines such as IL-1β, TNF-α or type I interferons [37].

More studies are required to assess if sLLT1 reflects ongoing (and past) systemic immune activation.

The demonstration of sLLT1 in patient sera raises the valid question if sLLT1 may have a putative functional role in the CD161-LLT1 interaction. Theoretically, sLLT1 may bind CD161 and as a consequence either block APC-T-cell interaction or signal via CD161. Although we cannot fully exclude a functional role for sLLT1 in RA, the available studies so far do not support a role for sLLT1 in CD161 binding. CLEC2D soluble isoforms failed to interact with CD161, leaving surface expressed LLT1 (isoform 1) as the sole ligand for this receptor [11].
